# Workplace Wellbeing in Ontario’s Public Sector: Employee and Employer Perspectives on Workplace Supports

**DOI:** 10.3390/ijerph23070909

**Published:** 2026-07-16

**Authors:** Heather Katherine Scott-Marshall, Tegan Slot, Lucas Marshall

**Affiliations:** 1 Social & Behavioural Health Sciences Division, Dalla Lana School of Public Health, University of Toronto, Toronto, ON M5T 3M7, Canada; 2 Mission Research, Toronto, ON M5C 1C4, Canada; lmarshall@missionresearch.ca; 3 Public Services Health & Safety Association (PSHSA), Toronto, ON M2N 6K1, Canada; tslot@pshsa.ca

**Keywords:** workplace wellbeing, workplace supports, occupational health, mental health, psychosocial risks, fatigue management, employee–employer perceptions, support availability, public sector

## Abstract

**Highlights:**

**Public health relevance—How does this work relate to a public health issue?**

- Workplace wellbeing is a public health issue because psychosocial working conditions, mental health, fatigue, and work–life balance can affect workers’ physical, psychological, and emotional wellbeing.
- This study examines workplace wellbeing in Ontario’s public sector, where employees provide essential services in healthcare, education, public safety, and government.

**Public health significance—Why is this work of significance to public health?**

- The findings identify consistent differences between employee and employer perspectives, with employees assigning greater importance and perceived effectiveness to many workplace wellbeing dimensions and supports.
- The study shows that workplace support availability does not consistently translate into use, particularly for mental health, psychological health and safety, and fatigue-related supports.

**Public health implications—What are the key implications or messages for practitioners, policy makers and/or researchers in public health?**

- Workplace wellbeing strategies should be designed around employee priorities and should address barriers to uptake, rather than focusing only on whether supports are formally available.
- Among employees who used at least one support, the positive association between cumulative support use and wellbeing suggests that integrated, system-level approaches may be more effective than isolated interventions, with fatigue management warranting particular attention.

**Abstract:**

Background: Workplace wellbeing has become a key focus of occupational and public health, with increasing attention to mental health, psychosocial risks, fatigue, and work–life balance. However, limited evidence compares employee and employer perspectives, distinguishes support availability from support use, or examines whether wellbeing is more closely associated with isolated interventions or the broader support environment. Method: Cross-sectional survey data were collected from public-sector employees (*N* = 1307) and employers (*N* = 321) in Ontario, Canada. Analyses compared employee and employer ratings of workplace wellbeing dimensions and supports, examined support availability and use, and assessed associations between support use and perceived workplace impact on personal wellbeing using linear regression models. Results: Employees consistently rated workplace wellbeing dimensions and supports as more important and more effective than employers. Several supports were widely available, but availability varied across sectors and did not consistently translate into use, particularly for mental health, psychological health and safety, and fatigue-related supports. Among employees using at least one support, the cumulative number of supports used was positively associated with wellbeing (B = 0.196, *p* < 0.01), whereas few individual supports demonstrated independent associations. Fatigue management programs showed a consistent positive association across model specifications. Conclusions: Findings suggest that workplace wellbeing is associated with the broader support environment, not only with individual programs. Employee-informed, integrated approaches that improve both access and uptake may strengthen workplace wellbeing; however, causal interpretation is limited by the cross-sectional design.

## 1. Introduction

The importance of workplace wellbeing has gained increasing prominence across organizational and policy contexts. In this study, workplace wellbeing refers to the perceived contribution of the work environment to employees’ physical, psychological, and emotional wellbeing. Recent frameworks, including CSA Z1003 and ISO 45003, reflect a broader shift in occupational health from a primary focus on physical safety to a more comprehensive approach that incorporates mental health, organizational culture, and psychosocial working conditions [1,2,3]. CSA Z1003 provides a Canadian standard for psychological health and safety in the workplace, while ISO 45003 provides international guidance on managing psychosocial risks within occupational health and safety management systems [1,3]. This shift has been further accelerated by structural changes in the nature of work, including increasing work intensity, digital connectivity, and evolving expectations regarding work–life boundaries [4,5,6].

The present study is informed by psychosocial risk, organizational climate, and systems-level approaches to workplace wellbeing. Psychosocial risk frameworks emphasize that wellbeing is shaped not only by individual resources, but also by work organization, job demands, autonomy, interpersonal relationships, leadership practices, and organizational policies [2,5,7]. Organizational climate theory similarly suggests that employees’ shared perceptions of policies, practices, and priorities influence how organizational supports are interpreted and used [8]. Total Worker Health and related systems-based approaches further emphasize the integration of health protection and health promotion, rather than reliance on isolated programs [9,10,11].

In the post-pandemic context, concerns related to burnout, disengagement, and employee retention have become particularly salient. Public discourse and emerging evidence point to sustained levels of employee strain and a re-evaluation of the role of work in daily life, with employees placing greater emphasis on balance, autonomy, and psychological wellbeing [12,13,14]. In response, organizations have expanded investments in workplace wellbeing initiatives aimed at supporting mental health, improving work environments, and enhancing employee engagement and performance [7,14,15,16].

Despite this increased attention and investment, three gaps remain important. First, organizations continue to implement a wide range of workplace wellbeing supports, yet there is limited clarity regarding which supports are most strongly associated with employee wellbeing [17,18]. Second, existing research rarely compares employee and employer perspectives, even though employer perceptions shape program design, prioritization, and resource allocation [16,19]. Third, relatively little is known about how the formal availability of supports translates into actual use, and whether access alone is sufficient to influence perceived wellbeing [20,21,22]. These gaps are particularly important in the public sector, where employees provide essential services and where wellbeing has implications for both worker health and service delivery. Understanding the relationship between perceived importance, perceived effectiveness, structural availability, and use is therefore critical for identifying where gaps emerge and how workplace wellbeing strategies can be more effectively targeted.

### Study Objectives

The present study examines workplace wellbeing across multiple dimensions within an Ontario public-sector context. The study was guided by four objectives: to compare employee and employer perspectives on the importance of workplace wellbeing dimensions and the perceived effectiveness of workplace supports; to assess the availability of supports across sectors; to evaluate use among employees with access; and to examine associations between workplace support use and perceived workplace impact on personal wellbeing. Given the independent, unmatched employee and employer samples and the exploratory nature of the analysis, these objectives are framed as research objectives rather than formal hypotheses.

## 2. Materials and Methods

### 2.1. Survey Design and Administration

The survey was developed collaboratively by the Public Services Health and Safety Association (PSHSA) and researchers from the University of Toronto’s Dalla Lana School of Public Health. A survey design was selected because the study aimed to compare perceptions across large employee and employer samples, assess availability and use of multiple workplace supports, and examine associations between support use and perceived wellbeing across public-sector settings. The manuscript was prepared with reference to the STROBE reporting guidelines for observational studies [23]. The survey was informed by established frameworks and prior research on workplace wellbeing and psychosocial working conditions [1,3,24] and included items on sociodemographic characteristics, sector of employment, job characteristics, and the availability and use of workplace wellbeing supports. Perceived importance was assessed for key workplace wellbeing dimensions, while perceived effectiveness was assessed for workplace wellbeing supports.

Parallel survey instruments were developed for employees and employers, with identical measures for key variables to enable direct comparison. The surveys were administered approximately one year apart: February–March 2024 for employees and January–February 2025 for employers. The employer and employee surveys were designed as independent population-based samples rather than matched employer–employee samples from the same workplaces. Comparisons therefore reflect differences between sampled employee and employer populations rather than within-workplace employee–employer dyads. Respondents were recruited through established opt-in online research panels maintained by external panel providers. Screening criteria were used to identify public-sector employees and employers in sectors served by PSHSA. The panel approach was intended to approximate the Ontario public-sector workforce served by PSHSA, but it should not be interpreted as a fully probability-based sample. Eligibility criteria were applied to ensure respondents were part of the approximately 1.6 million workers served by PSHSA (approximately 20% of the Ontario workforce aged 18–65). For the employer survey, respondents were restricted to organizations with at least 20 employees to increase the likelihood that formal workplace policies or programs were in place. Informed consent was obtained from all subjects involved in the study by the survey panel provider prior to participation. All data were anonymized prior to access by the research team, and analyses were conducted on aggregated data only.

### 2.2. Sample

The initial employer sample included 400 respondents. After excluding individuals who reported employment across multiple sectors, the final analytic sample comprised 321 employers. The employer sample size was selected to support descriptive comparison across the major public-sector groups served by PSHSA while remaining feasible for a specialized employer population.

The employee sample initially included 2141 respondents. To enhance comparability in working conditions, workplace exposures, sector assignment, and access to on-site workplace supports, the analytic sample was restricted to individuals in permanent (salaried or hourly) positions working primarily on-site within a single sector. Respondents in temporary, contract, seasonal, casual, or self-employed roles, as well as those working remotely or in hybrid arrangements, were excluded. This restriction improved comparability across respondents but limits generalizability to remote, hybrid, temporary, contract, seasonal, casual, and self-employed workers. The final analytic sample consisted of 1307 employees.

### 2.3. Measures

#### 2.3.1. Outcome

The primary outcome was perceived workplace impact on overall personal wellbeing. Respondents were asked: “Thinking about how workplace wellbeing can affect different aspects of your physical, psychological, and emotional wellbeing, how would you rate the impact of your current workplace on each of the following?” Responses were recorded on a 0–10 scale, where 0 indicated “extremely negative” and 10 indicated “extremely positive.” The item was designed to capture a global evaluative judgment of the perceived impact of the workplace on personal wellbeing. Internal consistency is not applicable to a single-item measure; however, single-item global measures can be appropriate when the construct is concrete, and the objective is to capture an overall evaluation rather than a multidimensional latent construct [25].

A composite wellbeing index was also constructed using additional items (general mood, work performance, sleep, physical health, social wellbeing, and health behaviors). Internal consistency was high (Cronbach’s alpha = 0.91), and this index was used in sensitivity analyses to assess whether findings were robust to an alternative, multi-item outcome specification.

#### 2.3.2. Importance of Workplace Wellbeing Dimensions

Perceived importance was assessed for 13 dimensions of workplace wellbeing using a 0–10 scale (0 = “not at all important,” 10 = “extremely important”). Dimensions included occupational health and safety, psychological health and safety, ergonomics, job satisfaction, work–life balance, diversity, equity, inclusion and accessibility (DEIA), interpersonal relationships, workload, financial security, recognition and rewards, influence over work processes, leadership interactions, and adequacy of resources.

#### 2.3.3. Effectiveness of Workplace Wellbeing Supports

Perceived effectiveness was assessed for 15 workplace wellbeing supports using a 0–10 scale (0 = “not effective at all,” 10 = “extremely effective”). Supports included physical fitness programs, mental health counseling, mental health awareness training, psychological health and safety programs, nutrition programs, health clinics, work–life balance programs, smoking cessation programs, team-building activities, ergonomics and safety programs, recognition programs, benefits packages, DEI initiatives, disability management programs, and fatigue management programs.

#### 2.3.4. Availability and Use of Supports

For each support, respondents indicated whether it was available at their workplace (yes/no). Employees also reported whether they had used each support (yes/no). Use was analyzed as conditional on reported availability.

### 2.4. Statistical Analysis

Data were analyzed using IBM SPSS Statistics (Version 29.0; IBM Corp., Armonk, NY, USA). Descriptive analyses were conducted to summarize sociodemographic and work-related characteristics of the sample, including age, gender, tenure, sector, union status, and organizational size. These analyses also included comparisons between employees and employers on perceived importance of workplace wellbeing dimensions and perceived effectiveness of workplace supports.

Employee–employer differences were assessed using independent samples *t*-tests across 13 wellbeing dimensions and 15 workplace supports. To account for multiple testing while retaining power across families of related comparisons, *p*-values were adjusted using the Benjamini–Hochberg false discovery rate procedure [26], and adjusted *p*-values (p_FDR) are reported. Full results are provided in Appendix A (Table A1 and Table A2).

Associations between workplace supports and perceived workplace impact on personal wellbeing were examined using linear regression. Analyses involving the wellbeing outcome and workplace support predictors were restricted to employees who reported current use of at least one workplace support (*n* = 1237). This restriction was applied because the primary objective of the regression analyses was to examine variation in wellbeing across patterns of support use among employees who had engaged with workplace wellbeing initiatives, rather than to estimate the association between any support use and no support use. However, this analytic decision may introduce selection bias because support users may differ systematically from non-users in awareness, perceived need, motivation, trust, workload, or workplace climate. Results from the regression models should therefore be interpreted as applying to employees already engaged with at least one workplace support, not to the full employee population. The outcome variable was treated as continuous (0–10 scale). Model assumptions were assessed using standard diagnostic procedures, including inspection of the outcome distribution and plots of standardized residuals versus predicted values. The outcome exhibited no substantial departures from approximate normality, and residual plots indicated no evidence of systematic non-linearity or heteroscedasticity, supporting the use of linear regression models. Multicollinearity was assessed using variance inflation factors (VIF) and tolerance statistics; all VIF values were low (approximately 1.07–1.14), and tolerance values were high (approximately 0.88–0.94), indicating no evidence of problematic multicollinearity.

A series of models were estimated: Model 1: demographic and employment covariates only; Model 2: total number of supports used; Model 3: domain-level composites (mental health supports; behavioral supports); Model 4: supports with ≥50 observed cases in the original (non-imputed) data, plus fatigue management; Model 5: all individual supports entered simultaneously. All models adjusted for age, gender, tenure, union status, organizational size, and sector.

#### 2.4.1. Handling of Missing Data

Missing data were addressed using multiple imputation, an established approach for reducing bias and loss of precision when data are incomplete under plausible missing-at-random assumptions [27]. Twenty imputed datasets were generated, and estimates were pooled using standard procedures. Imputation was applied to all variables included in the analytic models, including the outcome, workplace support variables, and covariates. Model coefficients and standard errors reflect pooled estimates. Adjusted R-squared values were calculated within each imputed dataset and averaged.

#### 2.4.2. Model Specification and Interpretation

The outcome variable was treated as continuous, consistent with prior research using 0–10 and Likert-type response scales in regression models. Unstandardized regression coefficients (B) and corresponding standard errors are reported. Statistical significance was evaluated using two-sided tests (*p* < 0.10, *p* < 0.05, *p* < 0.01, *p* < 0.001). Emphasis was placed on the direction, magnitude, and consistency of associations as well as statistical significance.

The modeling strategy assessed workplace supports at multiple levels of representation—cumulative exposure, domain-based groupings, and individual supports—to examine whether more integrated representations of workplace conditions provided greater explanatory power than models including individual supports alone. A prevalence threshold (≥50 cases in the original, non-imputed dataset) was applied in Model 4 to improve estimate stability and reduce the influence of sparse data.

## 3. Results

### 3.1. Sample Characteristics

Table 1 outlines the characteristics of the employee and employer samples. The employee sample (*N* = 1307) was predominantly female (64.6%), with just over half aged 35–54 (55.0%) and the remainder distributed across younger (24.3% aged 18–34) and older (20.7% aged 55+) age groups. Employees were relatively evenly distributed across sectors, with similar representation in healthcare (34.7%) and education (33.1%), followed by government (27.5%) and a smaller proportion in public safety (4.7%). The majority of employees were unionized (65.5%), had longer tenure (≥5 years; 55.7%), and worked in larger organizations (500+ employees; 67.7%).

The employer sample (*N* = 321), by contrast, was more likely to be male (55.5%) and more heavily concentrated in healthcare (48.9%) and public safety (20.9%), with lower representation from education (19.6%) and government (10.6%). Employers were also more likely to be unionized (83.7%), report shorter tenure (<5 years; 54.5%), and be drawn from smaller organizations (<500 employees; 60.1%).

### 3.2. Employee Wellbeing Outcomes

Table 2 presents descriptive statistics for employee-reported workplace impact on personal wellbeing and the composite wellbeing index, along with sector comparisons. Overall, employees reported moderately positive workplace impacts on personal wellbeing (M = 6.98, SD = 1.93), with mean scores ranging from 6.93 to 7.07 across sectors. A similar pattern was observed for the wellbeing index (M = 6.87, SD = 1.61). One-way ANOVA tests indicated no statistically significant differences across sectors for either outcome.

### 3.3. Perceived Importance of Workplace Wellbeing Dimensions

Table 3 presents employee–employer gaps in perceived importance across workplace wellbeing dimensions, overall and by sector. Differences were assessed using independent samples *t*-tests with Benjamini–Hochberg false discovery rate correction. Positive values indicate higher ratings among employees. Raw mean ratings are provided in Appendix A (Table A3).

Across all dimensions, employees rated workplace wellbeing factors as more important than employers. The largest gaps were observed for work–life balance (delta = 2.67), influence over work processes (delta = 2.62), and diversity, equity, inclusion, and accessibility (DEIA) (delta = 2.49), indicating substantial divergence in how these aspects of wellbeing are prioritized.

Moderate gaps were observed for interpersonal relationships, financial security, and job satisfaction, while the smallest difference was observed for occupational health and safety (delta = 0.29). This pattern suggests closer alignment on traditional physical safety issues than on psychosocial, relational, and equity-oriented dimensions of wellbeing.

The overall pattern was broadly consistent across sectors, although the magnitude of gaps varied. Gaps were often largest in healthcare, particularly for DEIA and work–life balance, whereas public safety tended to show smaller or more variable differences.

### 3.4. Perceived Effectiveness of Workplace Wellbeing Supports

Table 4 presents employee–employer gaps in perceived effectiveness of specific workplace wellbeing supports, overall and by sector. Across most supports, employees rated workplace wellbeing initiatives as more effective than employers. Raw mean ratings are provided in Appendix A (Table A4).

The largest gaps were observed for health insurance and compensation benefits (delta = 3.26), employee reward and recognition programs (delta = 2.75), and disability management programs (delta = 2.20). Moderate gaps were evident for team-based activities, ergonomics and safety programs, and DEIA initiatives, while smaller differences were observed for physical fitness programs, work–life balance programs, mental health counseling, mental health awareness, and smoking cessation programs.

Overall, these findings reinforce the pattern observed for perceived importance: employees tended to assign greater value to workplace wellbeing supports than employers, although the magnitude of difference varied by support and sector.

### 3.5. Availability of Workplace Supports

Table 5 presents the percentage of employees reporting that each workplace wellbeing support was available at their workplace.

Several supports were widely available across sectors, including health insurance and compensation benefits (93.9%), mental health counseling (85.4%), disability management programs (83.9%), and DEIA initiatives (81.7%). In contrast, health clinics (43.0%), physical fitness programs (40.7%), fatigue management programs (36.4%), and smoking cessation programs (27.1%) were less commonly available.

Sector differences were observed for several supports. Mental health counseling, DEIA initiatives, work–life balance programs, mental health awareness, and psychological health and safety programs varied by sector, as did health clinics. For other supports, availability was more similar across sectors.

Together, these findings indicate that some core supports are widely available, but access to several mental health, psychosocial, and work–life supports varies substantially across the public-sector context examined.

### 3.6. Use of Supports Among Those with Access

Table 6 presents the percentage of employees reporting use of each workplace wellbeing support among those indicating that the support was available.

Among employees with access, uptake was the highest for health insurance and compensation benefits (89.7%), team-based social activities (80.2%), employee recognition programs (62.5%), and health clinics (61.1%).

Moderate levels of use were observed for ergonomics programs, work–life balance programs, and DEIA initiatives, while lower uptake was evident for mental health counseling, fatigue management, disability management, and psychological health and safety programs. These patterns indicate that access alone does not ensure engagement, particularly for supports that may be sensitive, stigmatized, time-intensive, or dependent on trust in organizational confidentiality.

Sector differences in uptake were generally limited, although use of health insurance benefits (*p* = 0.040) and health clinics (*p* = 0.020) varied by sector, and smoking cessation programs approached statistical significance (*p* = 0.072).

### 3.7. Regression Analyses

To examine associations between the current use of workplace wellbeing supports and perceived workplace impact on personal wellbeing, a series of linear regression models were estimated among employees reporting use of at least one workplace support (*n* = 1237) (Table 7).

Across models, the cumulative number of supports used was positively associated with wellbeing (B = 0.196, *p* < 0.01), indicating that greater engagement with workplace supports corresponded to higher perceived wellbeing among employees who used at least one support. However, when supports were examined simultaneously, few demonstrated independent associations.

Fatigue management programs were a notable exception, showing a consistent positive association across all model specifications (B approximately 1.13–1.15, *p* < 0.01), with effect sizes exceeding one point on the 0–10 scale. This was the most robust individual-support finding in the regression analyses. Team building and social activities also demonstrated a consistent positive association across models (B approximately 0.49–0.51), although estimates were marginally significant.

At the domain level, neither behavioral nor mental health support composites were significantly associated with wellbeing. However, disaggregated analyses suggested heterogeneity within these domains: mental health awareness training showed a positive, trend-level association in the full model (B = 0.766, *p* < 0.10), whereas other mental health supports did not demonstrate consistent relationships.

Model fit improved with the inclusion of workplace supports, with adjusted R-squared increasing from 0.032 in the covariate-only model to 0.093 in the full model. This indicates that support-use variables explained a modest proportion of variance in perceived wellbeing, and that much of the variation likely reflects other individual, occupational, organizational, and social determinants not captured in the models.

Demographic and employment characteristics showed consistent associations with wellbeing across models. Older age was positively associated with wellbeing, while longer tenure (≥5 years) and unionized employment were associated with lower perceived workplace impact. Organizational size showed a negative but inconsistent association, and no statistically significant differences were observed across sectors.

### 3.8. Sensitivity and Interaction Analyses

As a sensitivity analysis, models were re-estimated using the composite wellbeing index. Results were broadly consistent with the primary analyses: fatigue management remained a strong predictor, while mental health awareness and team-based activities emerged as significant. Most supports remained non-significant, indicating that the overall pattern was robust to an alternative outcome specification.

Tests of interactions between sector and key supports (fatigue management, mental health awareness, and team-based social activities) were conducted; however, no statistically significant interactions were observed, indicating that these associations were consistent across sectors.

## 4. Discussion

This study examined workplace wellbeing across multiple levels: perceived importance, perceived effectiveness, and the structural availability and use of supports. It found consistent differences between employee and employer perspectives, variation in the availability and uptake of supports across sectors, and a positive association between cumulative support use and perceived wellbeing among employees who used at least one support. These findings are consistent with the view that workplace wellbeing is shaped by the broader support environment and organizational climate, rather than by isolated programs alone [8,9,10,11].

### 4.1. Employee–Employer Differences and Organizational Climate

The observed employee–employer differences have implications for how workplace wellbeing is conceptualized and implemented. The largest gaps were concentrated in domains related to work–life balance, autonomy, and DEIA, suggesting that employees placed greater emphasis on relational, structural, and equity-oriented aspects of work than employers. In contrast, closer alignment on occupational health and safety may reflect more established regulatory frameworks and clearer organizational accountability. From an organizational climate perspective, such differences matter because employees’ perceptions of priorities, practices, and support influence whether programs are viewed as credible, relevant, and safe to use [8].

### 4.2. Availability, Uptake, and the Overall Support Environment

A central finding was that formal availability did not consistently translate into use. This was particularly evident for mental health, psychological health and safety, disability management, and fatigue-related supports. Uptake may be shaped by awareness, perceived need, stigma, confidentiality concerns, manager support, workload, time constraints, trust in the organization, and whether supports are perceived as relevant or accessible [20,21,22,28]. The positive association between cumulative support use and wellbeing suggests that employees may experience supports as interconnected components of a broader supportive environment. This interpretation is consistent with systems-based approaches to workplace wellbeing, which emphasize the interaction of policies, practices, and working conditions rather than reliance on individual-level interventions alone [9,10,11,24].

### 4.3. Fatigue Management

Fatigue management programs emerged as the most consistent individual-support correlate of perceived wellbeing. This association should be interpreted cautiously because the cross-sectional design does not establish temporal ordering. Fatigue management may improve wellbeing by addressing workload, scheduling, recovery, and fatigue-related risk; alternatively, employees with better wellbeing may be more likely to access these programs, or organizations with stronger wellbeing climates may both offer fatigue programs and foster higher wellbeing. The finding may therefore reflect both the specific relevance of fatigue management and the broader maturity of an organization’s occupational health and wellbeing system. Given prior evidence linking fatigue to worker health and safety, particularly in safety-sensitive and care-oriented sectors, this finding warrants further longitudinal and implementation-focused research [29,30].

Taken together, the findings support an integrated, employee-informed approach to workplace wellbeing. They also indicate that associations between supports and wellbeing are modest and should not be interpreted as evidence that any single support independently improves wellbeing.

### 4.4. Study Limitations

Several limitations should be considered when interpreting these findings. The cross-sectional design precludes causal inference, and observed associations between workplace supports and wellbeing may reflect reverse causation or unmeasured confounding. For example, employees with higher wellbeing may be more likely to notice, access, or use supports, and organizations with stronger wellbeing climates may both provide more supports and foster higher wellbeing. All measures were self-reported, raising the possibility of common method bias, recall error, and social desirability effects. Measures of support availability and use were based on employee reports rather than organizational records, which may result in some misclassification of access.

The primary outcome was a single-item measure of perceived workplace impact on personal wellbeing. Although single-item global measures can be useful for capturing overall evaluative judgments [25], this item is not a validated diagnostic or multi-item wellbeing scale. The composite wellbeing index used in sensitivity analyses partially addresses this concern, but construct validity remains a limitation. The use of an external opt-in panel, while designed to approximate the public-sector workforce, may also limit representativeness and introduce selection bias. The analytic sample was restricted to primarily on-site, permanent employees to enhance comparability, but this limits generalizability to remote, hybrid, temporary, contract, seasonal, casual, and self-employed workers. Because the study was conducted within Ontario’s public sector and within sectors served by PSHSA, findings may not generalize to other provincial, national, or international jurisdictions; private-sector workplaces; smaller organizations; or public-sector organizations with different governance, workforce composition, collective bargaining structures, or wellbeing infrastructure. Organization type may also influence both the formal availability of supports and the workplace culture surrounding their use.

The employer and employee surveys were administered approximately one year apart and were not drawn from the same organizations. Accordingly, observed differences should be interpreted as differences between sampled employee and employer populations, not as direct evidence of within-organization employee–employer misalignment. Temporal changes in public discourse, organizational priorities, post-pandemic recovery, or workplace conditions may have influenced the observed differences. Some sector-specific subsamples, particularly in public safety, were relatively small, reducing statistical power to detect differences and interaction effects. The restriction of regression analyses to employees who used at least one support may introduce selection bias, as support users may differ systematically from non-users. Finally, the models explained a modest proportion of variance in perceived wellbeing, indicating that workplace supports represent only one component of a broader set of determinants.

### 4.5. Implications for Future Research

Findings from this study point to several directions for future research. Longitudinal and quasi-experimental designs are needed to better establish causal pathways between workplace supports and employee wellbeing, including the temporal sequencing of availability, uptake, and outcomes. Future studies should also examine mechanisms underlying the gap between availability and use, particularly for mental health, psychological health and safety, disability management, and fatigue-related supports. Qualitative research may be especially useful for understanding stigma, awareness, confidentiality concerns, workload constraints, manager support, and organizational culture.

The association between cumulative support use and wellbeing suggests value in examining workplace wellbeing as a system-level construct. Future work could explore composite or latent representations of support environments and their interaction with organizational climate. Given the emergence of fatigue management as a robust correlate, further research is warranted to unpack its components and identify which elements are most relevant for different employee groups and sectors. Evidence from prior research suggests that fatigue-related risks are particularly salient in healthcare and other safety-sensitive settings and may have broader implications for both worker and public safety [29,30].

Comparative studies across sectors, jurisdictions, and organization types, as well as analyses incorporating objective organizational data, would strengthen generalizability and reduce reliance on self-report. Future research should examine whether the availability, uptake, and perceived effectiveness of workplace wellbeing supports differ across provincial, national, and international contexts, private- and public-sector settings, small and large organizations, and organizations with different governance, collective bargaining, and wellbeing infrastructures. Future research should also incorporate diverse work arrangements and precarious employment contexts to capture a broader range of workplace experiences and support needs.

### 4.6. Policy and Practice Implications

The findings have several implications for policy and organizational practice. Differences between employee and employer perspectives highlight the need for systematic incorporation of employee voice in the design and prioritization of workplace wellbeing initiatives. Mechanisms such as participatory needs assessments, worker advisory groups, joint health and safety structures, and repeated employee feedback can help ensure that supports reflect employee priorities rather than only employer assumptions.

The gap between availability and use indicates that expanding access alone is insufficient. Organizations must also address barriers to uptake, including awareness, trust, stigma, confidentiality concerns, time constraints, manager support, and perceived relevance of supports [20,21,22,28]. The stronger association of cumulative supports with wellbeing underscores the value of integrated support environments rather than isolated initiatives.

The prominence of fatigue management suggests that interventions targeting workload, scheduling, recovery, and fatigue-related risk warrant particular attention within workplace wellbeing strategies, while recognizing that the present study demonstrates association rather than causation. Observed sector variation in availability also points to the need for targeted policy efforts in sectors where access to key supports is comparatively limited.

Taken together, these findings underscore the need for workplace wellbeing strategies that move beyond isolated interventions toward integrated, employee-informed approaches. Addressing differences in priorities, improving access, and reducing barriers to uptake may be especially important for supports related to mental health, psychological health and safety, disability management, and fatigue.

## 5. Conclusions

This study examined workplace wellbeing supports across multiple levels of analysis in an Ontario public-sector context. Findings indicate consistent differences between employee and employer perspectives, sector variation in support availability, and a gap between availability and use. Among employees using at least one support, cumulative support use was positively associated with perceived workplace impact on wellbeing, while few individual supports demonstrated independent associations. Fatigue management was a notable exception, showing a consistent positive association across models. Overall, the findings suggest that workplace wellbeing may be better understood as a function of the broader support environment than as the result of isolated interventions. Integrated, employee-informed strategies that align supports with employee priorities and reduce barriers to uptake may strengthen workplace wellbeing, although longitudinal research is needed to clarify causal pathways.

## Figures and Tables

**Table 1:** Characteristics of Employee and Employer Samples.

Characteristic	Employees	Employers
**Sex**		
Male	463 (35.4%)	178 (55.5%)
Female	844 (64.6%)	143 (44.5%)
**Age Group**		
18–34	318 (24.3%)	--
35–54	719 (55.0%)	--
55+	270 (20.7%)	--
**Sector**		
Healthcare	454 (34.7%)	157 (48.9%)
Education	432 (33.1%)	63 (19.6%)
Public Safety	61 (4.7%)	67 (20.9%)
Government	360 (27.5%)	34 (10.6%)
**Union Status**		
Not Unionized	451 (34.5%)	52 (16.3%)
Unionized	856 (65.5%)	269 (83.7%)
**Tenure**		
<5 years	579 (44.3%)	175 (54.5%)
5+ years	728 (55.7%)	146 (45.5%)
**Organization Size**		
<500 employees	422 (32.3%)	193 (60.1%)
500+ employees	885 (67.7%)	128 (39.9%)
**Total *N***	1307	321

**Table 2:** Descriptive Statistics and Sector Comparisons of Employee Wellbeing Outcomes.

Outcome Measure	Group	*n*	M	SD	SE	95% CI Lower	95% CI Upper	F (df)	*p*
Personal Wellbeing	Healthcare	454	7.00	1.97	0.09	6.81	7.18		
	Education	432	6.93	1.88	0.09	6.75	7.11		
	Public Safety	61	7.07	2.31	0.30	6.47	7.66		
	Government	360	7.00	1.88	0.10	6.80	7.20		
	Total	1307	6.98	1.93	0.05	6.88	7.09	F(3, 1234) = 0.14	0.933
Wellbeing Index	Healthcare	454	6.93	1.64	0.08	6.78	7.08		
	Education	432	6.88	1.49	0.07	6.74	7.03		
	Public Safety	61	6.80	1.93	0.25	6.31	7.30		
	Government	360	6.79	1.64	0.09	6.61	6.96		
	Total	1307	6.87	1.61	0.05	6.78	6.96	F(3, 1233) = 0.58	0.629

Note. F-tests are based on one-way ANOVA comparing sectors; no statistically significant differences were observed across sectors.

**Table 3:** Employer–Employee Gaps in Perceived Importance of Workplace Wellbeing Dimensions by Sector.

Dimension of Workplace Wellbeing	Overall (*N* = 1307/321)	Healthcare (*n* = 454/157)	Education (*n* = 432/63)	Public Safety (*n* = 61/67)	Government (*n* = 360/34)
Work–life balance	2.67 (#1) *	3.02 (#2) *	2.75 (#1) *	1.66 (#6) *	2.20 (#3)
Influence over work processes	2.62 (#2) *	2.70 (#4) *	2.66 (#2) *	2.53 (#1) *	2.66 (#1) *
Diversity, equity, inclusion and accessibility (DEIA)	2.49 (#3) *	3.10 (#1) *	2.35 (#5) *	2.32 (#2)	1.23 (#9)
Healthy interpersonal relationships with work peers	2.42 (#4) *	2.77 (#3) *	2.35 (#4) *	2.01 (#4) *	1.90 (#4)
Financial security and predictability	2.33 (#5) *	2.31 (#5) *	2.48 (#3) *	2.13 (#3)	2.42 (#2)
Job satisfaction and overall engagement with work	1.87 (#6) *	1.96 (#6)	2.11 (#7)	1.17 (#10)	1.82 (#5)
Psychological health and safety	1.57 (#7) *	1.75 (#7)	1.46 (#9)	1.57 (#9)	1.32 (#8)
Positive/constructive interactions with supervisors and leadership	1.38 (#8) *	0.92 (#10) *	2.10 (#8) *	1.57 (#7)	1.79 (#7)
Adequate resources to perform my job effectively	1.36 (#9) *	0.79 (#11) *	2.23 (#6) *	1.57 (#7)	1.81 (#6)
Ergonomically designed workplace	1.08 (#10)	1.40 (#8)	1.02 (#11)	0.61 (#11)	0.71 (#12)
Recognition and rewards programs	1.04 (#11)	1.14 (#9)	0.84 (#12)	1.90 (#5)	1.10 (#10)
Manageable amount of job tasks and workload	0.77 (#12) *	0.77 (#12) *	1.18 (#10)	−0.03 (#13)	1.08 (#11)
Occupational health and safety (physical hazards)	0.29 (#13) *	0.44 (#13) *	0.20 (#13)	0.43 (#12)	0.15 (#13)

Note. Ratings were provided on a 0–10 scale (0 = “not at all important,” 10 = “extremely important”). Values represent mean differences in perceived importance (employees minus employers); positive values indicate that employees rated a dimension as more important than employers. False discovery rate (FDR) correction was applied within each sector across the set of workplace wellbeing dimensions using the Benjamini–Hochberg procedure. Asterisks indicate statistical significance based on FDR-adjusted *p*-values (*p* < 0.05). Dimensions are ordered by overall mean difference (largest to smallest). Color intensity reflects the magnitude of employer–employee gaps within each sector (darker shading indicates larger gaps). Rank positions within sectors are shown in parentheses (#) for descriptive purposes only. Sample sizes are presented as employees/employers for each column (e.g., Overall *N* = 1307/321).

**Table 4:** Employer–Employee Gaps in Perceived Effectiveness of Wellbeing Supports by Sector.

Workplace Wellbeing Support	Overall (*N* = 1307/321)	Healthcare (*n* = 454/157)	Education (*n* = 432/63)	Public Safety (*n* = 61/67)	Government (*n* = 360/34)
Health insurance and other compensation benefits	3.26 (#1) *	3.23 (#1) *	3.17 (#1) *	2.37 (#1)	3.90 (#1) *
Employee reward and recognition programs	2.75 (#2) *	3.08 (#2) *	2.88 (#2) *	2.09 (#4)	3.16 (#2) *
Disability management and work accommodation programs	2.20 (#3) *	2.20 (#4) *	2.23 (#4) *	2.27 (#2) *	2.43 (#3) *
Team building and social activities	2.08 (#4) *	2.27 (#3)	2.46 (#3)	1.75 (#5)	2.17 (#5)
Ergonomics and workplace safety programs	2.04 (#5) *	2.20 (#5) *	2.01 (#6)	2.10 (#3)	1.75 (#6)
Diversity, equity, inclusion and accessibility (DEIA) programs	1.78 (#6) *	1.64 (#6) *	2.22 (#5) *	1.30 (#6)	2.38 (#4) *
Nutrition and healthy eating programs	1.24 (#7)	1.31 (#7)	1.38 (#7)	0.96 (#7)	0.98 (#8)
Physical fitness programs	0.91 (#8)	0.78 (#11)	1.25 (#8)	0.87 (#8)	0.92 (#9)
Work–life balance programs	0.87 (#9)	1.09 (#9)	1.13 (#10)	−0.38 (#15)	0.63 (#11)
Mental health counseling	0.86 (#10)	0.84 (#10)	0.68 (#12)	0.83 (#9)	1.25 (#7) *
Fatigue management programs	0.82 (#11)	1.18 (#8)	0.84 (#11)	0.59 (#10)	0.81 (#10)
Health clinics and check-ups	0.69 (#12)	0.57 (#13)	1.23 (#9)	0.56 (#11)	0.23 (#14)
Psychological health and safety programs	0.45 (#13)	0.63 (#12)	0.18 (#15)	0.44 (#13)	0.52 (#12)
Smoking cessation programs or workshops	0.28 (#14)	0.28 (#14)	0.47 (#13)	−0.16 (#14)	0.21 (#15)
Mental health awareness	−0.05 (#15)	−0.45 (#15)	0.38 (#14)	0.46 (#12)	0.38 (#13)

Note. Ratings were provided on a 0–10 scale (0 = “not at all effective,” 10 = “extremely effective”). Values represent mean differences in perceived effectiveness (employees minus employers); positive values indicate that employees rated a support as more effective than employers. False discovery rate (FDR) correction was applied within each sector across the set of workplace supports using the Benjamini–Hochberg procedure. Asterisks indicate statistical significance based on FDR-adjusted *p*-values (*p* < 0.05). Supports are ordered by overall mean difference (largest to smallest). Color intensity reflects the magnitude of employee–employer gaps within each sector (darker shading indicates larger gaps). Rank positions within sectors are shown in parentheses (#) for descriptive purposes only. Sample sizes are presented as employees/employers for each column (e.g., Overall *N* = 1307/321).

**Table 5:** Percentage of Employees Reporting Workplace Wellbeing Support as Available, by Sector.

Workplace Wellbeing Support	Omnibus *p* (Sector Differences)	Overall (*N* = 1307)	Healthcare (*n* = 454)	Education (*n* = 432)	Public Safety (*n* = 61)	Government (*n* = 360)
		% Yes (n)	% Yes (n)	% Yes (n)	% Yes (n)	% Yes (n)
Health insurance and other compensation benefits	ns	93.9% (1227)	92.6% (420)	93.9% (406)	91.6% (56)	95.9% (345)
Mental health counseling	<0.001	85.4% (1116)	76.7% (348)	89.0% (384)	62.5% (38)	95.0% (342)
Disability management and work accommodation programs	ns	83.9% (1097)	81.1% (368)	84.7% (366)	78.4% (48)	87.0% (313)
Diversity, equity, inclusion and accessibility (DEIA) programs	<0.001	81.7% (1068)	73.4% (333)	83.8% (362)	62.1% (38)	92.1% (332)
Ergonomics and workplace safety programs	0.072	73.0% (954)	73.3% (333)	60.8% (263)	74.1% (45)	86.9% (313)
Work–life balance programs	0.004	70.0% (915)	64.8% (294)	62.8% (271)	79.3% (48)	83.2% (300)
Mental health awareness	0.002	68.5% (895)	59.9% (272)	70.7% (305)	75.4% (46)	75.1% (270)
Psychological health and safety programs	0.001	65.5% (856)	58.3% (265)	65.0% (281)	69.8% (43)	73.8% (266)
Team building and social activities	0.010	61.6% (805)	58.0% (263)	60.2% (260)	40.2% (25)	71.0% (256)
Employee reward and recognition programs	ns	47.4% (620)	50.3% (228)	28.3% (122)	66.6% (41)	63.2% (228)
Health clinics and check-ups	<0.001	43.0% (562)	67.9% (308)	28.7% (124)	52.7% (32)	28.9% (104)
Physical fitness programs	ns	40.7% (532)	45.5% (207)	35.7% (154)	54.1% (33)	38.6% (139)
Fatigue management programs	ns	36.4% (476)	34.4% (156)	29.7% (128)	43.9% (27)	45.3% (163)
Nutrition and healthy eating programs	ns	33.1% (433)	35.0% (159)	32.0% (138)	42.1% (26)	30.8% (111)
Smoking cessation programs or workshops	ns	27.1% (354)	35.2% (160)	17.1% (74)	19.6% (12)	30.6% (110)

Note. Percentages reflect the proportion of employees reporting each support as available at their workplace. Omnibus *p*-values are from chi-square tests comparing availability across sectors; ns = not statistically significant. Supports are ordered by overall percentage (highest to lowest). Color intensity reflects percentage availability within each sector.

**Table 6:** Percentage of Employees Reporting Use of Workplace Wellbeing Support Among Those Reporting Support as Available, by Sector.

Workplace Wellbeing Support	Omnibus *p* (Sector Differences)	Overall (*N* = 1307)	Healthcare (*n* = 454)	Education (*n* = 432)	Public Safety (*n* = 61)	Government (*n* = 360)
		% Used (n/N available)	% Used (n/N available)	% Used (n/N available)	% Used (n/N available)	% Used (n/N available)
Health insurance and other compensation benefits	0.040	89.7% (1101/1227)	85.7% (360/420)	92.8% (377/406)	86.6% (48/56)	91.1% (314/345)
Team building and social activities	ns	80.2% (646/805)	79.3% (209/263)	82.8% (215/260)	71.4% (18/25)	79.7% (204/256)
Employee reward and recognition programs	0.053	62.5% (388/620)	55.6% (127/228)	64.4% (79/122)	61.4% (25/41)	68.5% (156/228)
Health clinics and check-ups	0.020	61.1% (343/562)	74.4% (229/308)	54.2% (67/124)	67.5% (22/32)	52.6% (55/104)
Ergonomics and workplace safety programs	ns	58.7% (560/954)	55.8% (186/333)	59.0% (155/263)	44.6% (20/45)	64.1% (201/313)
Work–life balance programs	ns	55.6% (509/915)	51.5% (151/294)	54.1% (147/271)	53.9% (26/48)	62.3% (187/300)
Diversity, equity, inclusion and accessibility (DEIA) programs	ns	55.5% (593/1068)	51.6% (172/333)	58.2% (211/362)	58.2% (22/38)	56.3% (187/332)
Physical fitness programs	ns	49.0% (261/532)	55.5% (115/207)	41.5% (64/154)	62.5% (21/33)	47.9% (67/139)
Nutrition and healthy eating programs	ns	45.0% (195/433)	46.0% (73/159)	41.0% (57/138)	65.9% (17/26)	45.1% (50/111)
Mental health awareness	ns	42.7% (382/895)	42.7% (116/272)	39.2% (120/305)	59.6% (27/46)	44.1% (119/270)
Mental health counseling	ns	38.6% (431/1116)	35.5% (124/348)	37.5% (144/384)	50.5% (19/38)	41.8% (143/342)
Fatigue management programs	ns	37.9% (180/476)	38.9% (61/156)	37.8% (48/128)	48.4% (13/27)	35.3% (58/163)
Disability management and work accommodation programs	ns	37.1% (407/1097)	35.3% (130/368)	37.4% (137/366)	56.8% (27/48)	35.9% (112/313)
Psychological health and safety programs	ns	31.3% (268/856)	31.1% (82/265)	34.5% (97/281)	50.9% (22/43)	24.6% (65/266)
Smoking cessation programs or workshops	0.072	25.5% (90/354)	33.0% (53/160)	21.2% (16/74)	55.7% (7/12)	16.8% (18/110)

Note. Column sample sizes (n) reflect total sector sample sizes; denominators for percentages are the number of employees reporting each support as available (N available), as shown in each cell. Percentages represent use among those with access. Omnibus *p*-values are from logistic regression models testing for overall sector differences in support use among employees reporting each support as available (models estimated separately for each support); ns = not statistically significant. Supports are ordered by overall percentage use (highest to lowest). Color intensity reflects percentage use, with darker shading indicating higher use.

**Table 7:** Linear Regression Models Predicting Workplace Impact on Personal Wellbeing Among Employees.

	Model 1—Covariates Only	Model 2—Cumulative Supports	Model 3—Support Domains (Composite Measures)	Model 4—High-Prevalence Supports (*n* ≥ 50)	Model 5—Full Model
**Demographics**					
Age	0.251 (0.110) *	0.312 (0.112) **	0.334 (0.128) *	0.313 (0.119) **	0.328 (0.132) *
Female	−0.189 (0.150)	−0.136 (0.154)	−0.093 (0.166)	−0.095 (0.158)	−0.104 (0.168)
**Employment characteristics**					
Tenure ≥ 5 years (ref = <5 years)	−0.386 (0.152) *	−0.366 (0.152) *	−0.355 (0.155) *	−0.358 (0.156) *	−0.335 (0.155) *
Unionized	−0.506 (0.161) **	−0.458 (0.164) **	−0.422 (0.173) *	−0.406 (0.171) *	−0.406 (0.173) *
Large organization (ref = small/medium)	−0.310 (0.159) †	−0.225 (0.168)	−0.213 (0.180)	−0.224 (0.171)	−0.245 (0.188)
**Sector**					
Sector: Healthcare	−0.190 (0.180)	−0.187 (0.181)	−0.103 (0.203)	−0.081 (0.197)	−0.109 (0.212)
Sector: Education	−0.377 (0.368)	−0.529 (0.384)	−0.324 (0.395)	−0.244 (0.387)	−0.382 (0.408)
Sector: Public Safety	−0.026 (0.178)	−0.010 (0.184)	0.070 (0.198)	0.098 (0.195)	0.080 (0.209)
**Workplace wellbeing supports**					
Number of workplace wellbeing supports (cumulative)		0.196 (0.062) **			
Health behavior supports (composite)			0.052 (0.266)		
Mental health supports (composite)			0.292 (0.273)		
Health clinics and check-ups			0.193 (0.287)	0.199 (0.291)	0.183 (0.287)
Work–life balance programs			0.359 (0.232)	0.382 (0.229) †	0.332 (0.235)
Team building and social activities			0.507 (0.266) †	0.509 (0.265) †	0.496 (0.268)†
Ergonomics and workplace safety programs			0.154 (0.218)	0.157 (0.219)	0.155 (0.222)
Employee reward and recognition programs			0.262 (0.274)	0.261 (0.273)	0.251 (0.271)
Health insurance and other compensation benefits			−0.001 (0.192)	−0.003 (0.197)	0.001 (0.191)
Diversity, equity, inclusion and accessibility (DEIA) programs			−0.052 (0.217)	−0.044 (0.219)	−0.055 (0.212)
Disability management and work accommodation programs			−0.326 (0.265)	−0.330 (0.263)	−0.307 (0.263)
Fatigue management programs			1.148 (0.370) **	1.153 (0.372) **	1.125 (0.364) **
Physical fitness programs					0.157 (0.273)
Mental health counseling					−0.311 (0.393)
Mental health awareness training					0.766 (0.435) †
Psychological health and safety programs					0.452 (0.384)
Nutrition and healthy eating programs					0.068 (0.378)
Smoking cessation programs or workshops					−0.030 (0.459)
*n*	1237	1237	1237	1237	1237
Adjusted R^2^	0.032	0.056	0.084	0.079	0.093

† *p* < 0.10, * *p* < 0.05, ** *p* < 0.01. Notes: Linear regression models predicting perceived workplace impact on personal wellbeing (0–10 scale). Coefficients represent unstandardized estimates with standard errors in parentheses. Analyses were restricted to employees reporting current use of at least one workplace support (*n* = 1237). Reference categories are male, non-unionized, small/medium organizations, and government sector. Mental health supports combine mental health counseling, awareness training, and psychological health and safety programs; behavioral supports combine nutrition, smoking cessation, and physical fitness programs. Model 4 includes supports with at least 50 cases in the original (non-imputed) data; fatigue management programs were additionally retained due to consistently strong associations across models. Adjusted R^2^ values are averaged across imputed datasets.

## Data Availability

The data that support the findings of this study are not publicly available due to restrictions related to the use of third-party survey panel data.

## Appendix A. Supplementary Analyses

### Appendix A.1. False Discovery Rate Adjustments

This appendix presents supplementary statistical results supporting the main analyses. Table A1 and Table A2 report Benjamini–Hochberg false discovery rate (FDR)-adjusted *p*-values for tests of differences in perceived importance of workplace wellbeing dimensions and perceived effectiveness of workplace wellbeing supports, respectively. These results are provided to ensure transparency in the handling of multiple comparisons. Patterns were broadly consistent across sectors; therefore, sector-specific results are not presented separately.

**Table A1:** Benjamini–Hochberg false discovery rate (FDR) adjustment for perceived importance of workplace wellbeing dimensions.

Dimension	Raw *p*-Values	FDR-Adjusted *p*-Value
Work–life balance	0.000	0.000
Diversity, equity, inclusion and accessibility (DEIA)	0.000	0.000
Healthy interpersonal relationships with work peers	0.000	0.000
Influence over work processes	0.000	0.000
Positive/constructive interactions with supervisors and leadership	0.000	0.000
Adequate resources to perform my job effectively	0.000	0.000
Occupational health and safety (physical hazards)	0.002	0.004
Job satisfaction and overall engagement with work	0.002	0.003
Financial security and predictability	0.006	0.009
Manageable amount of job tasks and workload	0.007	0.009
Psychological health and safety	0.008	0.009
Ergonomically designed workplace	0.109	0.118
Recognition and rewards programs	0.151	0.151

Note. False discovery rate (FDR) correction was applied using the Benjamini–Hochberg procedure across the set of 13 workplace wellbeing dimensions. Values below 0.05 are considered statistically significant after FDR correction. Sector-specific patterns were broadly consistent with the overall results.

**Table A2:** Benjamini–Hochberg false discovery rate (FDR) adjustment for perceived effectiveness of workplace wellbeing supports.

Support	Raw *p*-Values	FDR-Adjusted *p*-Value
Employee reward and recognition programs	0.000	0.000
Health insurance and other compensation benefits	0.000	0.000
Diversity, equity, inclusion and accessibility (DEIA) programs	0.000	0.000
Disability management and work accommodation programs	0.000	0.000
Ergonomics and workplace safety programs	0.003	0.009
Team building and social activities	0.015	0.038
Nutrition and healthy eating programs	0.032	0.069
Physical fitness programs	0.067	0.126
Work–life balance programs	0.084	0.140
Health clinics and check-ups	0.376	0.470
Mental health counseling	0.214	0.292
Mental health awareness programs	0.866	0.866
Psychological health and safety programs	0.604	0.663
Smoking cessation programs or workshops	0.619	0.663
Fatigue management programs	0.115	0.173

Note. False discovery rate (FDR) correction was applied using the Benjamini–Hochberg procedure across the set of 15 workplace wellbeing supports. Values below 0.05 are considered statistically significant after FDR correction. Sector-specific patterns were broadly consistent with the overall results.

### Appendix A.2. Descriptive Statistics (Mean Ratings)

Table A3 and Table A4 present employee and employer mean ratings (with standard errors) for perceived importance of workplace wellbeing dimensions and perceived effectiveness of workplace wellbeing supports, respectively. These tables are provided to support interpretation of the mean differences reported in Table 3 and Table 4.

**Table A3:** Employee and Employer Mean Ratings of Importance Across Workplace Wellbeing Dimensions.

	Employees (*N* = 1307)	Employers (*N* = 321)
Workplace Wellbeing Dimension	M (SE)	M (SE)
Occupational health and safety (physical hazards)	8.5974 (0.05102)	8.3063 (0.08101)
Psychological health and safety	8.5909 (0.06540)	7.0178 (0.53868)
Ergonomically designed workplace	8.2579 (0.07150)	7.1761 (0.65720)
Job satisfaction and overall engagement with work	8.7484 (0.06096)	6.8825 (0.50446)
Work–life balance	9.0221 (0.06459)	6.3561 (0.41719)
Diversity, equity, inclusion and accessibility (DEIA)	7.7522 (0.10067)	5.2664 (0.59477)
Healthy interpersonal relationships with work peers	8.8197 (0.07152)	6.4002 (0.38683)
Manageable amount of job tasks and workload	8.8416 (0.06921)	8.0680 (0.26072)
Financial security and predictability	9.0642 (0.06463)	6.7350 (0.76095)
Recognition and rewards programs	7.4456 (0.09835)	6.4041 (0.69361)
Influence over work processes	8.2719 (0.07618)	5.6566 (0.42523)
Positive/constructive interactions with supervisors and leadership	8.9146 (0.06219)	7.5356 (0.32764)
Adequate resources to perform my job effectively	8.9378 (0.05199)	7.5741 (0.32055)

Note. Ratings were provided on a 0–10 scale (0 = “not at all important,” 10 = “extremely important”). Values are presented as means with standard errors in parentheses (M [SE]). Sample sizes reflect respondents included in the analytic sample (employees *N* =1307; employers *N* = 321). Means and standard errors are pooled across imputed datasets. Mean differences and statistical tests are presented in Table 3.

**Table A4:** Employee and Employer Mean Ratings of Effectiveness Across Workplace Wellbeing Supports.

	Employees (*N* = 1307)	Employers (*N* = 321)
Workplace Wellbeing Supports	M (SE)	M (SE)
Physical fitness programs	7.5487 (0.08237)	6.6406 (0.45004)
Mental health counseling	7.5461 (0.07867)	6.6877 (0.66351)
Mental health awareness	7.2218 (0.09971)	7.2716 (0.28177)
Psychological health and safety programs	7.2300 (0.08968)	6.7791 (0.85801)
Nutrition and healthy eating programs	7.0412 (0.08649)	5.8034 (0.55435)
Health clinics and check-ups	7.3864 (0.10067)	6.6919 (0.74297)
Work–life balance programs	8.1171 (0.09193)	7.2516 (0.47344)
Smoking cessation programs or workshops	5.8840 (0.14227)	5.6061 (0.57631)
Team building and social activities	7.0440 (0.08916)	4.9673 (0.75977)
Ergonomics and workplace safety programs	7.5285 (0.07578)	5.4903 (0.62234)
Employee reward and recognition programs	7.3814 (0.09645)	4.6361 (0.48080)
Health insurance and other compensation benefits	8.7856 (0.06904)	5.5258 (0.42317)
Diversity, equity, inclusion and accessibility (DEIA) programs	6.6076 (0.10119)	4.8261 (0.40939)
Disability management and work accommodation programs	7.5643 (0.07769)	5.3619 (0.32402)
Fatigue management programs	7.0053 (0.09655)	6.1891 (0.52195)

Note. Ratings were provided on a 0–10 scale (0 = “not at all effective,” 10 = “extremely effective”). Values are presented as means with standard errors in parentheses (M [SE]). Sample sizes reflect respondents included in the analytic sample (employees *N* = 1307; employers *N* = 321). Means and standard errors are pooled across imputed datasets. Mean differences and statistical tests are presented in Table 4.
